# Using Artificial Intelligence to Predict the Development of Kyphosis Disease: A Systematic Review

**DOI:** 10.7759/cureus.48341

**Published:** 2023-11-06

**Authors:** Yehia Y Hussein, Muhammad Mohsin Khan

**Affiliations:** 1 General Practice, Hamad Medical Corporation, Doha, QAT; 2 Neurosurgery, Hamad Medical Corporation, Doha, QAT

**Keywords:** spine surgery, ai & robotics in healthcare, kyphosis, new technology in spine surgery, ai and machine learning

## Abstract

The use of artificial intelligence in the field of medicine - including spine surgery - is now widespread and prominent. Kyphosis is a prevalent disease in spine surgery with abundant morbidity. Predicting the development of kyphosis disease has been somewhat difficult, and the use of AI to aid in the prediction of kyphosis disease may yield new opportunities for spine surgeons. The aim of this review is to recognize the contributions of AI in predicting the development of kyphosis. Five databases/registers were searched to identify suitable records for this review. Nine studies were included in this review. The studies demonstrated that AI could be utilized to predict the development of kyphosis disease after corrective surgery for a variety of spinal pathologies, including thoracolumbar burst fracture, cervical deformity, previous kyphosis disease, and adult degenerative scoliosis. The studies utilized a variety of AI modalities, including support vector machines, decision trees, random forests, and artificial neural networks. Two of the included studies also compared the use of different AI modalities in predicting the development of kyphosis disease. The literature has demonstrated that AI can be utilized effectively to predict the development of kyphosis disease. However, the current research is limited and only sparsely covers this broad field. Therefore, we suggest that further research is needed to explore the uncharted opportunities in predicting the development of kyphosis disease. Also, further research would confirm and consolidate the benefits demonstrated by the literature included in this review.

## Introduction and background

Twenty years ago, the study of artificial intelligence (AI) was an uncharted field that was explored only by pioneers. Nowadays, the term artificial intelligence is becoming more widely known and its applications are becoming more prominent and more widespread, especially in scientific fields. Artificial intelligence is the reproduction of human intelligence using computational power. There are many definitions for artificial intelligence, but Russell and Norvig (2010) have categorized the defining features of artificial intelligence into four categories: the ability to think humanly, the ability to think rationally, the ability to behave humanly, and the ability to behave rationally [1]. Moreover, they have gauged the success of artificial intelligence based on its comparability to human performance and its rationality.

The field of medicine has already seen extensive use of artificial intelligence. Many researchers have already utilized artificial intelligence to predict the development of common diseases such as heart disease [2-5] and diabetes [6]. Researchers have also experimented with the application of artificial intelligence in spine surgery in a wide array of problems [7,8]. For example, some researchers have demonstrated the automation of artificial intelligence in localization and segmentation of the spine on CT and MRI scans [9,10]; moreover, researchers have used artificial intelligence to predict the progression of malalignment after deformity correction surgery in adolescent idiopathic scoliosis [11]. Some researchers have also used artificial intelligence to predict vertebrae at risk of insufficiency fractures [12].

Kyphosis is an anteriorly convex curvature of the spine. Slight kyphosis is normal in the thoracic region, but the cervical and lumbar spine’s normal alignment is lordosis (anteriorly concave curvature of the spine), and a kyphotic curvature in the cervical and/or lumbar spine is pathologic.

Pathologic kyphosis is a major cause of morbidity in spine surgery. The most common cervical spine deformity is kyphosis, and it is associated with a considerable reduction in quality of life [13,14]. Although cervical kyphosis can be managed surgically with spine deformity correction, the durability of the correction is variable and revision rates exceed 20% due to a variety of postoperative complications, especially junctional kyphosis [15-17]. Lumbar kyphosis is also a significant cause of morbidity as low back pain is one of the most common presenting complaints in primary care [18,19], and it is widely accepted that spinal malalignment (e.g., lumbar kyphosis) is a major etiologic factor for lumbar back pain [20].

The involvement of AI in medicine has been extensive and it has been used proficiently in the field of spine surgery. Furthermore, recognizing and appreciating the utility of AI in predicting the development of kyphosis may be tremendously advantageous as it would allow us to effectively predict more accurately which patients are more likely to develop kyphosis disease. The aim of this paper is to review the current literature to recognize the contributions of AI in predicting the development of kyphosis and to discuss the benefits and limitations of using AI to predict the development of kyphosis disease.

## Review

Methods

The authors performed a comprehensive systematic review complying with the Preferred Reporting Items for Systematic Reviews and Meta-Analyses (PRISMA) guidelines [21]. A literature search was conducted on July 5, 2023, using six electronic databases: Cumulative Index to Nursing and Allied Health Literature (CINAHL), Cochrane, MEDLINE, PubMed, Scopus, and Academic Search Ultimate. Google Scholar was also used to identify additional records. The searches were performed using the following search terms ((“Artificial intelligence”) OR (“Machine learning”)) AND (“Spine”) AND (“Kyphosis”). The search was executed in the aforementioned databases, and 480 records were identified. Two independent investigators (YH, MK) conducted a manual screening of the records’ titles and abstracts based on their relevance and the selection criteria. The inclusion criteria were (1) the study population is adults above the age of 18, (2) it involves the use of AI to predict the development of kyphosis, (3) it was published between 1/1/2015 and 5/7/2023, (4) it is in the English language. The exclusion criteria were (1) the study population includes pediatric patients, (2) not predicting kyphosis (e.g., prediction of scoliosis or other malalignments), (3) not using artificial intelligence to predict kyphosis, (4) does not provide new information (e.g., narrative review), (5) is published before 31/12/2014, (6) is published in a non-English language.

**Figure 1:**
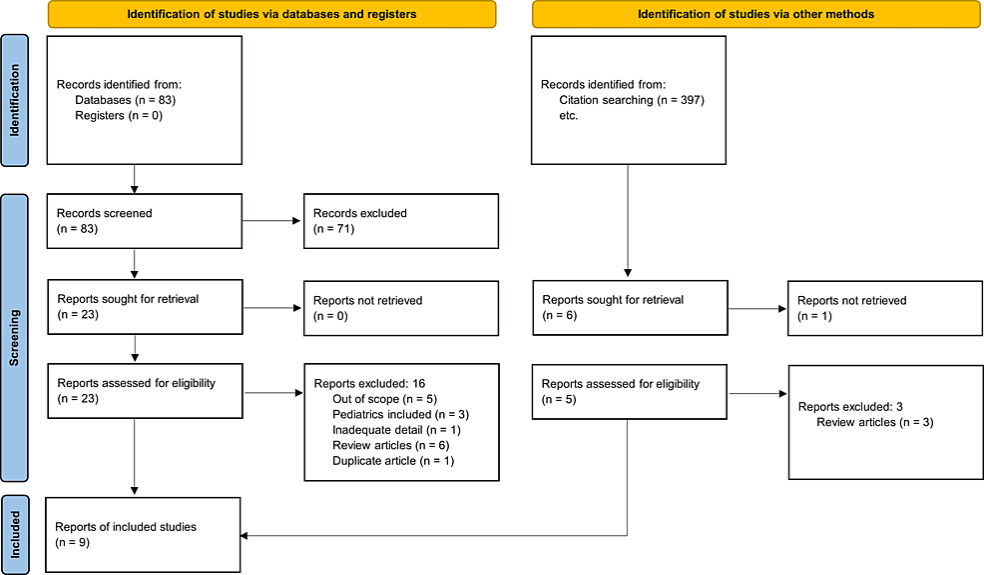
PRISMA Diagram demonstrating the selection process PRISMA: Preferred Reporting Items for Systematic Reviews and Meta-Analyses

After discussing and evaluating their screened studies, both investigators agreed on 28 records to assess thoroughly for eligibility. A full-text assessment of the 28 records was performed and both investigators agreed to exclude 19 after rigorous application of the selection criteria. This resulted in nine studies being included in this systematic review. The processing of data is summarized in Figure 1. Quality assessment was performed using the Strengthening the Reporting of Observational Studies in Epidemiology (STROBE) criteria [22] since all the selected studies were observational studies.

Results

A summary of the nine selected studies is presented in Table 1. Only one study investigates the use of AI to predict kyphosis in spinal fracture patients. Two studies involve predicting kyphosis after cervical deformity correction surgery. Two studies investigate the prediction of kyphosis in adult spinal deformity patients, and two other studies investigate the prediction of kyphosis after corrective surgery for adult spinal deformity. One study investigated the prediction of kyphosis postoperatively in patients who underwent corrective spine surgery for kyphosis problems. One study investigates the prediction of kyphosis in adult degenerative or idiopathic scoliosis.

**Table 1:** Summary of included studies using AI to predict the development of kyphosis disease AI: Artificial intelligence

Study	Journal	Study Design	Pathology	Use of AI	Aim of Study	Findings	STROBE Points	STROBE Score
Dong et al., 2022 [23]	BMC Musculoskeletal Disorders	Case-control	Thoracolumbar burst fracture	Support Vector Model (SVM)	To identify predictors poor postoperative alignment of the thoracolumbar region after PPSF in patients with TLBF using a support vector machine (SVM) model	The variables associated with poor postoperative alignment were intervertebral disc injury (42%), surgically corrected Cobb angle (25%), preoperative Cobb angle (18%), and IPD (15%).	1,2,3,4,5,7,8,9,10,11,13,14,15,16,17,18,19,20,21,22	19
Dankwa & Zheng, 2019 [24]	MDPI	Retrospective cohort	Corrective spine surgery	Random forest (RF), support vector machine (SVM), artificial neural network (ANN)	To predict the development of kyphosis disease using ML algorithms in patients who have undergone corrective spine surgery	ANN (3-6-6-1) model outperformed all the other models	1,2,3,4,5,8,11,12,13,14,15,16,17,18,19,20,22	17
Passias et al., 2018 [25]	The Spine Journal	Case-control	Cervical deformity surgery	Random forest (RF) & decision trees (DT)	To develop a risk index for the development of DJK in the first postoperative year	The most relevant clinical predictor of DJK was the presence of neurologic symptoms. No significant relationship was found between osteoporosis, age, nor ambulatory status, and the incidence of DJK. Baseline radiographic malalignments were the strongest predictors for DJK.	1,2,3,4,5,6,7,8,9,11,13,14,15,16,17,18,19,20,22	19
Passias et al., 2022 [26]	Spine	Retrospective cohort	Cervical deformity surgery	Conditional Inference Tree (CIT)	To investigate the impact of postoperative radiographic alignment on development of DJK in ACD patients.	Postoperative radiographic alignment is strongly associated with distal junctional kyphosis.	1,2,3,4,5,6,7,8,9,10,11,12,13,14,15,16,17,18,19,22	20
Scheer et al., 2016 [27]	Spine	Retrospective cohort	Adult Spinal Deformity	Decision trees (DT)	To build a model based on baseline demographic, radiographic, and surgical factors that can predict clinically significant proximal junctional kyphosis (PJK) and proximal junctional failure (PJF)	The best model produced had an overall model accuracy of 86.3% with an AUC of 0.89.	1,2,3,4,5,6,7,8,9,10,11,12,13,14,15,16,17,18,19,20,21,22	22
Lafage et al., 2023 [28]	Journal of Neurosurgery Spine	Cross-sectional	Adult Spinal Deformity	Cluster Analysis	To use an unsupervised cluster approach to identify patterns of adult spinal deformity and evaluate associated perioperative outcomes	Patients were classified into four clusters of deformity patterns: hyper-thoracic kyphosis, severe coronal, severe sagittal, and moderate sagittal. The 4 clusters differed in their perioperative outcomes, including pain scores, disability scores, functional impairment scores and osteotomies per case.	1,3,4,5,7,8,9,10,11,12,13,14,15,16,17,18,22	17
Lee et al., 2020 [29]	Global Spine Journal	Prospective Cohort	Corrective surgery for adult spinal deformity	Medicrea	To test whether a machine-learning (ML) program can predict postoperative thoracic kyphosis of the uninstrumented thoracic spine and pelvic compensation in patients who undergo fusion from the lower thoracic spine (T10 or T11) to the sacrum	The AI-predicted pelvic tilt and thoracic kyphosis correlated well with the actual postoperative values (uninstrumented TK: R^2^ = 0.764, P < 0.001; PT: R^2^ = 0.868, P < 0.001)	1,2,3,4,5,7,8,11,12,13,14,17,18,19,20,21,22	17
Chauhan et al., 2023 [30]	MDPI	Retrospective cohort	Corrective surgery for Kyphosis problems	Logistic Regression (LR), Naive Bayes (NB), Random Forest (RF), K-Nearest Neighbors (KNN), Support Vector Machine (SVM), and Deep Neural Network (DNN)	To determine the AI modality that cam best predict Kyphosis disease using biomedical data.	The Hyperparameter-tuned DNN models excelled over the other models. The DNN models’ accuracy was 87.72% with 5-fold cross-validation and 87.64% with 10-fold cross-validation.	1,2,3,15,16,17,18,19,20,21,22	11
Durand et al., 2021 [31]	European Spine Journal	Retrospective cohort	Adult degenerative or idiopathic scoliosis	Artificial neural network (ANN)	To use an artificial intelligence to cluster preoperative lateral radiographs into groups with distinct morphologies and identify any distinct perioperative outcomes	The ANN succeeded in clustering cases into 6 clusters. The relationship between sagittal vertical axis (SVA) and proximal junctional kyphosis difered by cluster.	2,3,4,5,6,8,11,12,13,14,15,16,17,18,19,21,22	17

The studies also employ a variety of artificial intelligence modalities, including support vector model (SVM), random forest (RF), decision trees (DT), conditional inference trees (CIT), artificial neural networks (ANN), and deep neural networks (DNN). These modalities were employed in a supervised and/or unsupervised manner.

Of the nine studies selected for inclusion in this review, seven of the studies leveraged AI modalities to predict the development of kyphosis disease and identify risk factors for the development of kyphosis disease. On the other hand, two studies were testing which AI modality was the best to predict kyphosis disease - and thus the investigators were utilizing the same input into each AI modality and testing to see which AI modality could best utilize the input to predict kyphosis disease.

Quality assessment

Quality assessment was performed using the STROBE checklist [22]. This is a 22-point checklist with a maximum score of 22. We used the STROBE guidelines because they are designed for the quality assessment of observational studies. After selection of the studies, we discovered all the studies were observational (cohort, case-control, or cross-sectional). After using the STROBE checklist for assessment, almost all of the included studies were of adequate quality with an average of 17.6. Most of the studies lacked quality in explaining how they recruited and followed-up participants (STROBE 6 & 13) to the study and how they addressed bias (STROBE 9); nevertheless, all the included studies were of high quality and only two studies were of relatively lower quality with scores of 11 & 12.

Discussion

The nine studies included in this review have demonstrated that artificial intelligence can be wielded effectively and flexibly to predict the development of kyphosis disease in many surgically treated and untreated spinal pathologies, including spinal fractures, degenerative spine disease, cervical deformity, adult spinal deformity, and corrective surgery for previous kyphosis disease.

The main advantage of using artificial intelligence to predict the development of kyphosis disease is its ability to predict the development of kyphosis using radiological parameters that are not traditionally used to predict the development of kyphosis; for example, Dong et al. [23] demonstrated that a support vector model was able to identify multiple variables associated with the development of postoperative kyphosis disease in patients who underwent percutaneous pedicle screw fixation after thoracolumbar burst fracture with favorable predictive performance, and the identified variables included intervertebral disc injury, surgically corrected Cobb angle, preoperative Cobb angle, and intervertebral distance. Predicting the development of postoperative kyphosis disease is difficult, and these are not parameters traditionally used to predict the development of postoperative kyphosis disease, so it is very advantageous that artificial intelligence can identify relevant variables and utilize these variables to predict the development of kyphosis disease. This highlights the benefit of machine learning (ML) in surgical planning and patient management in spinal fracture patients. However, this study is limited to burst fractures of the thoracolumbar spine; therefore, more studies are needed to confirm the effectiveness of AI in predicting the development of kyphosis disease in different types of spinal fractures (e.g., Chance fracture) in different segments of the spine.

Research displaying the prowess of artificial intelligence modalities in effectively predicting the development of kyphosis is essential, but it is equally important that researchers investigate which artificial intelligence modality is most effective in predicting kyphosis disease. Two of the selected studies explore the effectiveness of the different modalities. Dankwa and Zheng [24] investigated the effectiveness of a random forest, support vector machine, and artificial neural network, and the study concluded that a 3-6-6-1 artificial neural network (ANN (3-6-6-1)) was the most effective modality. However, the study was limited in that it only input the age, number of vertebrae involved, and the number of the topmost vertebrae operated on. Other relevant parameters, such as gender, height, degree of preoperative alignment, and radiologic images, were not utilized by the AI, and the output of the AI (kyphosis) was a binary variable - which doesn’t account for the varying possible degrees of kyphosis. Moreover, the generalizability of the study results is limited since they only studied patients who underwent “corrective spine surgery” and they did not specify the preoperative pathologies involved.

Chauhan et al. [30] also explored the effectiveness of different AI modalities. However, this was unfortunately the lowest quality study (STROBE 11) included in our systematic review. The study aims to utilize AI to identify which patients will have persistent kyphosis after corrective surgery; however, the AI only has the subjects’ ages, number of vertebrae involved, and position of the vertebrae involved. The AI has no information regarding the original pathology, neither the preoperative alignment nor the presence of comorbidities. Although the study concluded that the hyperparameter-tuned DNN was the most proficient AI modality at predicting kyphosis disease, the superficial initial input leaves room for doubt on whether the hyperparameter-tuned DNN would be as proficient if more detailed data was input. Nevertheless, the research's recommendation to use the DNN model for detecting and foreseeing Kyphosis disease after a clinical procedure demonstrates a significant step towards personalized medicine and disease detection, and such research should motivate scientists to conduct further research and utilize the benefits of artificial intelligence in predicting kyphosis disease.

As previously highlighted, seven of the nine selected studies examined the proficiency of AI in predicting the development of kyphosis disease under specific circumstances (e.g., using random forest and decision trees to predict the development of distal junctional kyphosis in patients undergoing cervical deformity correction surgery), and all seven studies demonstrated that the utilized AI modality was effective in predicting the development of kyphosis disease, which is concordant with the high level of effectiveness AI has demonstrated in the wider field of spine surgery [7,8]. However, the number of studies involved is limited and sparsely covers a wide array of possibilities; for example, there are no studies about the use of AI to predict the development of kyphosis disease in cervical fracture patients. The sparsity of studies is understandable given the novelty of the AI modalities.

At the time of writing this paper (August 2023) the United States Food and Drug Administration (U.S. FDA) AI/ML database shows 521 AI-enabled devices approved for use in medical practice [32]. However, the majority of these devices are used in the field of radiology and none of them are used to predict the development of kyphosis disease. This emphasizes the need for further studies investigating the applicability, safety, and effectiveness of specific AI modalities (e.g., ANN) in predicting the development of kyphosis disease.

Collectively, these studies contribute to a broader understanding of the role of AI and ML in spinal care. The success the studies displayed in predicting kyphosis through various machine learning models suggests a bright future for AI and ML in spinal care. The convergence of AI and spinal care is an exciting frontier that holds great promise for enhancing patient outcomes and advancing the field of spinal deformity studies. However, it is essential to recognize that the application of AI and ML in healthcare is still in its nascent stages. Challenges related to data privacy, ethical considerations, and potential biases must be addressed to harness the full potential of these technologies. Collaborative efforts between clinicians, researchers, and technologists are vital to ensure that AI and ML are implemented responsibly and effectively.

The limitations of this systematic review are that the number of studies is limited and the studies cover topics sparsely. The number of studies included in this review is only nine, and each study covers a unique situation - so each publication studies the use of a specific AI modality to predict the development of kyphosis disease in a specific pathology. Consequently, we were unable to conduct any meta-analysis of effect sizes since the studies included were not studying the same AI-pathology combinations.

## Conclusions

Predicting the development of kyphosis disease has remained somewhat elusive until recently, and the efficient use of artificial intelligence has demonstrated promising results so far. However, abundant research is needed to cover this broad and uncharted field of medicine. The current review highlights the current opportunities possible with the use of AI in predicting kyphosis disease, but more research is needed to document the unexplored opportunities. Although some authors have suggested that physicians begin using the investigated AI modality to predict kyphosis disease (e.g., using the hyperparameter-tuned DNN to predict the persistence of kyphosis disease after corrective surgery), we believe such recommendations are premature and unsafe as the use of such equipment, before further research and testing, may cause significant harm. However, we believe that further research and testing may definitely be able to confidently document the safety of such powerful tools in the prediction of kyphosis disease.
